# Mental health disparities by sex: unravelling determinants and changes in the refugee resettlement process over a decade

**DOI:** 10.1017/S2045796026100638

**Published:** 2026-04-07

**Authors:** Xinyan Bu, Meng Zheng, Andre M.N. Renzaho, Thomas P. Nguyen, Shameran Slewa-Younan, Shiyu Feng, Xuezhi Liang, Wen Chen

**Affiliations:** 1 Department of Medical Statistics, School of Public Health, Sun Yat-sen University, Guangzhou, China; 2 Center for Migrant Health Policy, Sun Yat-sen University, Guangzhou, China; 3 Mental Health, School of Medicine, Western Sydney University, Sydney, NSW, Australia; 4 Department of Psychiatry, Austin Hospital, University of Melbourne, Melbourne, VIC, Australia; 5 Clinical Sciences, Murdoch Children's Research Institute, Melbourne, VIC, Australia; 6 Translational Health Research Institute, School of Medicine, Western Sydney University, Campbelltown, NSW, Australia; 7 Centre for Mental Health, Melbourne School of Population and Global Health, University of Melbourne, Melbourne, VIC, Australia

**Keywords:** evidence-based psychiatry, Fairlie decomposition, gender differences, mental health, PTSD, refugees

## Abstract

**Aims:**

Significant sex disparities in mental health have been observed amongst resettled refugees, yet how these disparities and their determinants evolve over time remains unclear. This study sought to quantitatively unravel determinants and changes in mental health disparities by sex.

**Methods:**

Data were drawn from Waves 1 (2013–2014), 5 (2017–2018) and 6 (2023) of the 10-year *Building a New Life in Australia* (BNLA) cohort. Post-traumatic stress disorder (PTSD) and high risk of severe mental illness (HR-SMI) were measured using the PTSD-8 and Kessler-6 scales. Fairlie method was used to quantify the disparity (total predicted probability difference by sex) and the contribution proportion of individual determinants (explained difference/total predicted probability difference × 100%).

**Results:**

A total of 2261 refugees were included at Wave 1, with 1833 (81.1%) and 905 (40.0%) followed up at Waves 5 and 6. Female refugees consistently experienced poor mental health, with the total predicted probability difference decreasing from the initial (Wave 1, 8.3%) to middle stage (Wave 5, 4.6%), then increasing in the long term (Wave 6, 6.3%). Determinants of disparities varied across waves, but poor status of physical health was a persistent contributor of disparities in PTSD (contribution proportion: 57.2%, 71.5% and 63.0% at each wave). Family conflict contributed at the initial (HR-SMI: 4.5%) and long-term stages (PTSD: 8.7%), while financial hardships (PTSD: 13.2%; HR-SMI: 23.2%), marital status (HR-SMI: 24.8%) and family concerns (PTSD: 8.0%) were key determinants at the middle stage. Unmet support or help during COVID-19 was a major contributor at Wave 6 (PTSD: 22.7%; HR-SMI: 8.0%).

**Conclusions:**

Sex disparities exist in refugees’ mental health and require sustained attention and tailored strategies. To promote mental health equity, there is a long-term need to provide essential physical healthcare and financial assistance and address family-related stressors. Additionally, it is important to identify and address the specific psychosocial needs of women in times of crisis such as the COVID-19 pandemic.

## Introduction

By the end of 2024, the number of forcibly displaced people worldwide rose to an unprecedented high of 123.2 million, with 42.7 million of those individuals being refugees (UNHCR, 2025). Refugees are defined as those who have been forced to leave their homelands due to experiences such as persecution, conflict and human rights violations. Owing to the significant adversities and experiences of potentially traumatic events encountered throughout their migration journeys, refugees have higher prevalence rates of post-traumatic stress disorder (PTSD), depression and anxiety when compared to non-refugee populations living in conflict or war settings (Henkelmann *et al.*, 2020). These effects may be compounded by pre-existing physical health problems as well as resettlement-related stressors such as loneliness, family separation, financial hardships and language barriers (Steel *et al.*, 2011; Mulugeta *et al.*, 2019; Acarturk *et al.*, 2021; Nguyen *et al.*, 2024). During the early phase of resettlement, refugees have been consistently found to have higher levels of PTSD, depression and psychological distress than the host population (Steel *et al.*, 2009; Handiso *et al.*, 2025). Over time, multiple longitudinal studies have reported a gradual decline in symptoms of PTSD and psychological distress in resettlement countries as refugees gain access to settlement services such as social support, employment and language services (Steel *et al.*, 2009; Silove *et al.*, 2017). However, complex non-linear trajectories have also been reported, with refugees demonstrating an initial decline in symptoms during early resettlement periods followed by a later worsening which has been postulated to be related to cumulative stress or unresolved trauma (Silove *et al.*, 1997; Montgomery, 2010; Bogic *et al.*, 2012; Li *et al.*, 2016). Thus, the hypothesis that decreasing resettlement-related stressors account for improvements in mental health morbidity remains uncertain given the mixed evidence to date (Handiso *et al.*, 2025).

Recent research on mental health trajectories among resettled refugees has also examined the impact of major global events such as the COVID-19 pandemic, with emerging evidence suggesting that such events may disrupt established trajectories and are associated with increases in PTSD and psychological distress from pre-pandemic to post-pandemic in refugees resettled in Australia (Zheng *et al.*, 2025). Notably, this study also reported significantly higher rates of high psychological distress among female participants compared to males (Zheng *et al.*, 2025). This pattern aligns with findings from population-based, non-refugee samples showing consistently higher rates of PTSD and other common mental disorders in females (Kessler *et al.*, 2012; Viertiö *et al.*, 2021). It has been argued that multi-level interactions among biology, psychology and sociology, as articulated using a biopsychosocial lens (Engel, 1977; Bolton, 2023), may account for such differences. That is sex-linked variations in stress physiology may increase females’ vulnerability to mental illnesses such as PTSD, but these biological factors must be understood alongside females’ disproportionate exposure to psychosocial adversities, for example, sex-based violence, thus highlighting the central role psychosocial determinants play in shaping mental health risk (Olff *et al.*, 2007).

Within refugee populations, evidence for sex disparities in PTSD and other common mental disorders among refugee populations is also emerging with studies reporting a higher symptom burden among females (Chung *et al.*, 1998; Bogic *et al.*, 2015; Tinghög *et al.*, 2017; Ainamani *et al.*, 2020; Blackmore *et al.*, 2020; Acarturk *et al.*, 2021). For example, a community-based cross-sectional study of Southeast Asian refugees found higher psychological distress among females and demonstrated sex-specific predictors, such that multiple traumatic events and older age were found to predict distress for both sexes, whereas males were additionally affected by low income and limited English proficiency and females’ distress was further associated with lower educational attainment in their region of origin and fewer years residing in the United States (Chung *et al.*, 1998). When examining predictors of PTSD, similar to general population cohorts, interpersonal violence appears particularly salient for females. In a cross-sectional study of Congolese refugees in Uganda, females reported higher PTSD prevalence and stronger dose–response effects particularly following rape, and a systematic review similarly identified sexual trauma as a consistent contributor to elevated PTSD risk in female refugees (Ainamani *et al.*, 2020; Vallejo-Martín *et al.*, 2021). Despite these findings, most studies to date have been cross-sectional, thus precluding any causative conclusions and explanations of directionality.

By contrast, only a few longitudinal studies have examined whether determinants confer risk among refugee males and females. Handiso et al. found that females had higher odds of developing PTSD than males if they experienced financial hardship, unemployment or were in short-term lease housing (Handiso *et al.*, 2024). Meanwhile, for psychological distress, females were only at higher odds if they had experienced one to four financial hardships whereas males had higher odds of elevated psychological distress if they reported five to six financial hardships (Handiso *et al.*, 2024). Another secondary data analysis of the same longitudinal study similarly found that financial hardship was more saliently related to PTSD in females compared to males (Wu *et al.*, 2021). For males, problems with adjusting to life in Australia were more saliently related to PTSD and psychological distress compared to females (Wu *et al.*, 2021). Despite the valuable insights gained from these studies, our understanding of the determinants of these sex-based mental health disparities remains limited. According to the Giddens’ Structuration theory, structures (rules, resources, institutions) shape individual action whilst agency (what people do, resist, adapt) simultaneously reproduces or transforms those structures. Such a lens suggests that the determinants of females’ mental health risks may shift, accumulate or be reconfigured across different stages of resettlement (Giddens, 1984). To our knowledge, no study to date has examined how determinants of sex disparities in mental health evolve over the course of the resettlement process or has quantified how much each determinant contributes to these differences over time. Also, little research to date has robustly characterized the impact of the COVID-19 pandemic on these sex disparities.

To address these gaps in the literature, we drew on three waves of the Building a New Life in Australia (BNLA) study, a nationally representative longitudinal cohort study of refugees resettled in Australia over a 10-year period. By examining whether sex disparities in mental health outcomes emerge and persist during the first decade of refugee resettlement and by quantifying the key determinants underpinning these disparities, this study aims to generate critical insights needed to develop targeted, equitable and trauma-informed mental health interventions for refugee communities.

## Methods

### Study design and data source

Data for this longitudinal study were derived from a national resettled refugee cohort study, namely the BNLA study, aiming to better understand factors that influence resettlement outcomes. Baseline data were collected from October 2013 to March 2014 (Wave 1), followed by four annual waves (Waves 2–5), with Wave 5 conducted between October 2017 and March 2018. An additional wave (Wave 6) was conducted from January to July 2023, during the post-pandemic period, resulting in two approximately 5-year intervals: Waves 1–5 and Waves 5–6. To capture sex disparities in mental health at different resettlement stages, this study used data from Waves 1, 5 and 6, which were conducted in the first (initial stage), fifth (middle stage) and tenth years (long-term stage) of resettlement, respectively.

### Participants and procedure

**Figure 1.**
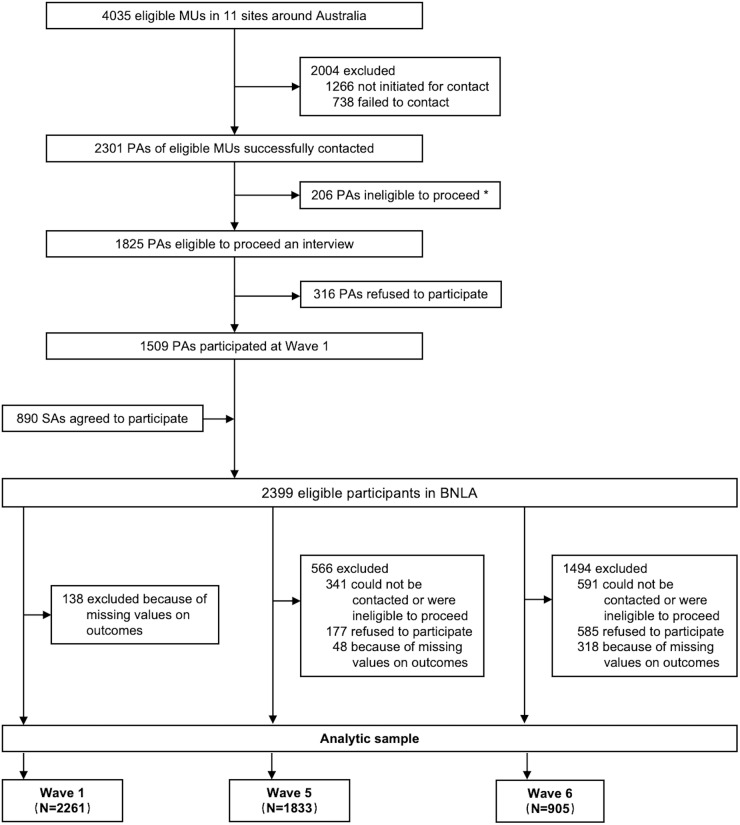
Flowchart. MUs: migration units. PAs: principal applicants. SAs: secondary applicants. *Ineligible for proceed: Successfully contacted, but unable to conduct the interview due to reasons such as quota fulfilment, relocation beyond the interview area, or unavailability during the fieldwork period.

BNLA participants were recruited from 11 research sites across Australia which had the largest number of refugees initially settled between November 2010 and October 2011. With a required sample size of 1500 migrant units (MUs, refugees sharing a visa were included in one MU), a census approach was used in each site to maximize the pool of eligible participants. Specifically, the eligible MUs in each site were identified by the Australian Department of Immigration and Border Protection. Eligibility for the study required that permanent humanitarian visas were granted between May and December 2013 (within 6 months prior to recruitment), and that principal applicants (PAs) were aged 18 or older. Then, the PAs of eligible MUs were contacted and invited to participate. Once PAs agreed to participate, secondary applicants who were 15 years or older in the same MU were invited to voluntarily participate. Participants with missing outcome measures were excluded from the analytical sample (Fig. 1).

Extensive consultations were undertaken by the Australian Institute of Family Studies (AIFS) to conceptualize and design the BNLA project. The project collected data on family composition, demographics, education and training, immigration experience, health and life in Australia. Four interview modes were applied: computer-assisted self-interviews (CASI), computer-assisted personal interviews (CAPI), computer-assisted telephone interview (CATI) and computer-assisted web interview (CAWI). At Waves 1 and 5, the survey was administered as a face-to-face interview via CASI (70.5% at Wave 1; 45.0% at Wave 5) or CAPI (29.5% at Wave 1; 50.0% at Wave 5). At Wave 5, CATI interviews (4.9%) were used for respondents who were unavailable for face-to-face interviews. At Wave 6, the primary mode was CAWI (52.1%), with CAPI (30.5%), CASI (11.9%) and CATI (5.6%) offered based on respondent preference. To account for the diverse cultural backgrounds of participants, with the availability of interpreters, 19, 10 and 7 languages were used across the Wave 1, Wave 5 and Wave 6 samples, respectively. Additional details about the BNLA project are provided by the Australian Department of Social Services (Australian Institute of Family Studies, 2024).

### Measurements

#### Outcome variables

PTSD symptoms over the past week were assessed by the PTSD-8 scale, which covers all three symptom clusters of the DSM-IV PTSD diagnosis: intrusion, avoidance and hyperarousal (Kleijn *et al.*, 2001). Each item on the PTSD-8 is rated on a 4-point Likert scale (ranging from ‘not at all’ to ‘most of the time’). Respondents were classified as having PTSD if they answered ‘sometimes’ or ‘most of the time’ on at least one item in each of the three PTSD symptom clusters. The PTSD-8 scale demonstrated excellent internal consistency in the BNLA project, with a Cronbach’s alpha of 0.92, 0.95 and 0.95 at Waves 1, 5 and 6, respectively.

High risk of severe mental illness (HR-SMI) over the past 4 weeks was measured by the Kessler Screening Scale for Psychological Distress (K6) scale, which includes six items that measure the presence of nervousness, hopelessness, irritability, negative affect, fatigue and worthlessness (National Comorbidity Survey, 2003). The K6 uses a 5-point Likert scale, ranging from ‘none of the time’ (1) to ‘all of the time’ (5), with total scores ranging from 6 to 30. Based on the cut-off point recommended by the Australian Bureau of Statistics, participants with a K6 score of 19 or higher were classified as having HR-SMI. The K6 scale exhibited strong internal consistency in the BNLA project, with a Cronbach’s alpha of 0.89, 0.92 and 0.92 at Waves 1, 5 and 6, respectively.

### Stratified variables

Analyses were stratified by sex (male or female) as the BNLA study only collected data on binary biological sex.

### Potential determinants

Using prior research in the area of refugee mental health (Cooper *et al.*, 2019; Henkelmann *et al.*, 2020; Atrooz *et al.*, 2024; Nguyen *et al.*, 2024), individual demographic characteristics, pre-settlement adversities, physical health-related factors, resettlement-related stressors and COVID-19-related factors were included as potential determinants of mental health disparities. Individual demographics included age (≤34, 35–49, 50–64 or ≥65), education attainment (below undergraduate or tertiary degree, undergraduate and tertiary degree), marital status (single, married/cohabitating), region of origin (North Africa and the Middle East, South-East Asia, Southern and Central Asia, or Sub-Saharan Africa) and number of financial hardships (0, 1, 2, ≥3) experienced since arriving in Australia at Wave 1 or in the past 12 months at subsequent waves (Torlinska *et al.*, 2020).

Physical health-related factors included self-rated overall health (very poor-fair, good-excellent), difficulty in daily work due to physical health (not at all-somewhat, quite a lot and above) and amount of bodily pain (none-mild, moderate-severe) (Dowling *et al.*, 2019).

Pre-settlement adversities included events that refugees had experienced before coming to Australia, including traumatic events (yes or no), refugee camp events (yes or no) and detention events (yes or no). Detention events were derived from two binary items: whether the participant had spent any time in immigration detention or community detention. If any of these items were answered with ‘yes’, the value for the detention events was recorded as ‘yes’; otherwise, it was recorded as ‘no’.

Resettlement-related stressors were measured with a self-report checklist about whether any of the following were a source of stress in their life in Australia: (1) loneliness stressor; (2) social integration stressors (including getting used to life in Australia and language barriers); (3) family conflicts in Australia; (4) school or study stressors and (5) concerns about family in Australia (including caring for family, family health and family safety).

Two COVID-19-related factors were additionally included at Wave 6 to account for the pandemic effect on sex disparities. COVID-19 stressor (yes/no) was assessed by asking whether the pandemic caused stress in life. Unmet support or help during COVID-19 was assessed using the question: ‘Overall, during the coronavirus restriction/lockdown periods in 2020 and 2021, how often did you feel that you needed support or help but could not get it from anyone?’ Responses were recorded on a 4-point ordinal scale: never, rarely, sometimes and often-very often.

### Statistical analysis

Participants’ characteristics and the prevalence of mental illness within sex-based subgroups were described using frequencies, and subgroup differences were examined by chi-square tests. Fairlie decomposition analyses were then conducted for each wave to capture mental health disparities by sex and their determinants at different resettlement stages.

To quantify the disparity between male and female refugees (males were set as the reference group) and attribute the disparity to each determinant in a quantitative approach, Fairlie method, a nonlinear Blinder–Oaxaca decomposition technique, was applied (Fairlie, 2005). Specifically, the total predicted probability difference in mental illness, namely the sex disparity, was derived from the difference in mean predicted probabilities for females and males estimated using a logistic regression model which included all potential determinants that were previously mentioned. For categorical predictors, the first category was designated as the reference category. Then, the twofold Fairlie method decomposed the disparity into two explained and unexplained parts. The explained part represents the endowment effects, that is, the disparity attributable to group differences in the distribution of observed determinants. The unexplained part captures disparity from group differences in the distribution of unobserved determinants and the coefficient effects. Consistent with Fairlie’s original formulation, which highlights the inherent difficulty in interpreting the unexplained part, our analysis focused on quantifying the endowment effects. The contribution of each individual determinant was assessed by contribution proportion (endowment effect/total predicted probability difference × 100%). The contribution proportion represents the share of the total predicted probability difference that can be attributed to adjusting the distribution of a given determinant to that of the reference group (males), while holding the distributions of the other determinants constant. The sign of the contribution proportion indicates the direction of impact on disparities: a positive value suggests the disparity would decrease by the share indicated by its contribution proportion if the determinant distribution were adjusted to that of the reference group, while a negative value implies it would increase. In essence, this analysis allows quantifying the percentage of the disparity by each determinant through the magnitude of its contribution proportion, and to determine, through its sign, whether that determinant ultimately widens or narrows the gap. The sum of the contribution proportion of all included determinants (overall contribution proportion) may exceed 100% in some cases, indicating that eliminating all between-group differences in included determinants would reverse the direction of the observed disparity (Isong *et al.*, 2018). We provide specific explanation cases in the results section. One thousand replications were performed with random orders of variables included in the regression analysis in each replication, so that contribution proportion estimates were not sensitive to ordering of variables.

Cross-sectional weights provided in the BNLA dataset (ranging from 0.37 to 2.50) were incorporated in the Fairlie method to adjust for the representativeness of the sample by adjusting participants’ sex, visa sub-class, capital city, age and region of origin information. Little’s Missing Completely at Random (MCAR) test and comparisons between complete and missing datasets were conducted to identify the missing data mechanism, indicating a missing at random (MAR) mechanism at Waves 1 and 5 and a missing completely at random mechanism at Wave 6. Therefore, missing data in explanatory variables were addressed using multiple imputation with the chained equations approach, and sensitivity analysis with complete data was conducted to test the robustness. Additionally, unweighted multiple imputation analyses were performed to assess the impact of sample weights. All statistical analyses were conducted using STATA (version 15.1) with a significance level of 0.05.

## Results

Figure 1 shows the flowchart of this study. A total of 2399 participants were included in the BNLA cohort at Wave 1, of whom 1881 and 1223 participants were successfully followed up at Waves 5 and 6, respectively. After excluding participants with missing values on outcomes, the analytic sample included 2261 (1028 females and 1233 males; Wave 1), 1833 (867 females and 966 males; Wave 5) and 905 (430 females and 475 males; Wave 6) eligible participants across three surveys conducted over a span of 10 years. Males had higher attrition rates from the BNLA cohort than females (23.9% vs. 18.9% at Wave 5, 51.3% vs. 46.3% at Wave 6). The distributions of a few characteristics were significantly different between males and females who were lost to follow-up (Supplementary materials 2), but similar distribution differences were also observed in the analytic sample.

Table 1 shows the unweighted prevalence of PTSD and HR-SMI in female refugees were significantly higher than those in males across all three waves (for PTSD, 37.7% vs. 29.8% at Wave 1, 31.8% vs. 25.5% at Wave 5, 35.3% vs. 28.2% at Wave 6; for HR-SMI, 22.3% vs. 12.7% at Wave 1, 19.3% vs. 15.1% at Wave 5, 27.9% vs. 16.8% at Wave 6; all *p* < 0.05). Across waves, there were no significant differences between males and females in age or loneliness stressor (*p* > 0.05). However, compared to males, females reported lower educational attainment (85.4% of females vs. 81.7% of males had below undergraduate education at Wave 1, *p* = 0.020), lower rates of being married/co-habiting (51.2% vs. 63.2% at Wave 1, *p* < 0.001) and a lower proportion of having experienced one or more detention events and refugee camps events (all *p* < 0.05 at Waves 1, 5 and 6). More females came from North Africa and the Middle East than males (59.6% vs. 53.4% at Wave 1, *p* = 0.001). A larger proportion of females reported poor overall health and experienced difficulties in daily work due to physical health, bodily pain (all *p* < 0.05 at Waves 1, 5 and 6), financial hardships (62.1% vs. 70.6% never had any financial hardship at Wave 5, *p* = 0.001), school or study-related stressors (all *p* < 0.05 at Waves 1, 5 and 6), family concern stressors (64.1% vs. 70.8% never reported at Wave 5, *p* = 0.009) and family conflicts in Australia (3.4% vs. 1.9% and 7.0% vs. 3.2% experienced ≥1 family conflict events at Waves 1 and 6, *p* = 0.030 and *p* = 0.013, respectively). Notably, more females reported unmet support or help during the COVID-19 pandemic (36.3% vs. 47.9% never reported, *p* = 0.006).

**Table 1.** Characteristics and prevalence of mental illness in sex-based subgroups of the BNLA participants at three survey waves

	Wave 1				Wave 5				Wave 6			
	Total (*N* = 2261)	Male (*N* = 1233)	Female (*N* = 1028)	*p*	Total (*N* = 1833)	Male (*N* = 966)	Female (*N* = 867)	*p*	Total (*N* = 905)	Male (*N* = 475)	Female (*N* = 430)	*p*
HR-SMI, *n* (%)				<0.001				0.022				<0.001
0 = No	1875 (82.9)	1076 (87.3)	799 (77.7)		1520 (82.9)	820 (84.9)	700 (80.7)		705 (77.9)	395 (83.2)	310 (72.1)	
1 = Yes	386 (17.1)	157 (12.7)	229 (22.3)		313 (17.1)	146 (15.1)	167 (19.3)		200 (22.1)	80 (16.8)	120 (27.9)	
PTSD, *n* (%)				<0.001				0.003				0.025
0 = No	1505 (66.6)	865 (70.2)	640 (62.3)		1311 (71.5)	720 (74.5)	591 (68.2)		619 (68.4)	341 (71.8)	278 (64.7)	
1 = Yes	756 (33.4)	368 (29.8)	388 (37.7)		522 (28.5)	246 (25.5)	276 (31.8)		286 (31.6)	134 (28.2)	152 (35.3)	
Age (years), *n* (%)				0.877				0.413				0.190
≤34	1118 (49.4)	611 (49.6)	507 (49.3)		687 (37.5)	346 (35.8)	341 (39.3)		212 (23.4)	98 (20.6)	114 (26.5)	
35–49	736 (32.6)	394 (32.0)	342 (33.3)		657 (35.8)	351 (36.3)	306 (35.3)		317 (35.0)	172 (36.2)	145 (33.7)	
50–64	315 (13.9)	177 (14.4)	138 (13.4)		351 (19.1)	191 (19.8)	160 (18.5)		262 (29.0)	140 (29.5)	122 (28.4)	
≥65	92 (4.1)	51 (4.1)	41 (4.0)		138 (7.5)	78 (8.1)	60 (6.9)		114 (12.6)	65 (13.7)	49 (11.4)	
Education, *n* (%)				0.020				0.009				0.092
0 = below undergraduate or tertiary degree	1874 (83.4)	999 (81.7)	875 (85.4)		1535 (84.5)	787 (82.3)	748 (86.9)		739 (82.7)	376 (80.5)	363 (85.0)	
1 = undergraduate and tertiary degree	373 (16.6)	224 (18.3)	149 (14.6)		282 (15.5)	169 (17.7)	113 (13.1)		155 (17.3)	91 (19.5)	64 (15.0)	
Marital status, *n* (%)				<0.001				<0.001				<0.001
0 = single	956 (42.3)	454 (36.8)	502 (48.8)		619 (33.8)	270 (28.0)	349 (40.3)		256 (28.3)	99 (20.8)	157 (36.5)	
1 = married or co-habiting	1305 (57.7)	779 (63.2)	526 (51.2)		1214 (66.2)	696 (72.0)	518 (59.7)		649 (71.7)	376 (79.2)	273 (63.5)	
Region of origin, *n* (%)				0.001				0.083				0.084
1 = North Africa and the Middle East	1271 (56.2)	658 (53.4)	613 (59.6)		1088 (59.4)	562 (58.2)	526 (60.7)		501 (55.4)	247 (52.0)	254 (59.1)	
2 = South-East Asia	124 (5.5)	61 (4.9)	63 (6.1)		96 (5.2)	44 (4.6)	52 (6.0)		64 (7.1)	32 (6.7)	32 (7.4)	
3 = Southern and Central Asia	779 (34.5)	469 (38.0)	310 (30.2)		591 (32.2)	331 (34.3)	260 (30.0)		312 (34.5)	182 (38.3)	130 (30.2)	
4 = Sub-Saharan Africa	81 (3.6)	44 (3.6)	37 (3.6)		55 (3.0)	29 (3.0)	26 (3.0)		27 (3.0)	14 (2.9)	13 (3.0)	
5 = Other	6 (0.3)	1 (0.1)	5 (0.5)		3 (0.2)	0 (0.0)	3 (0.3)		1 (0.1)	0 (0.0)	1 (0.2)	
Self-rated overall health, *n* (%)				<0.001				<0.001				0.002
1 = very poor-fair	851 (37.6)	381 (30.9)	470 (45.7)		728 (39.7)	332 (34.4)	396 (45.7)		393 (43.4)	183 (38.5)	210 (48.8)	
2 = good-excellent	1410 (62.4)	852 (69.1)	558 (54.3)		1105 (60.3)	634 (65.6)	471 (54.3)		512 (56.6)	292 (61.5)	220 (51.2)	
Amount of bodily pain, *n* (%)				<0.001				<0.001				0.001
0 = none-mild	1623 (71.8)	966 (78.3)	657 (63.9)		1195 (65.2)	678 (70.2)	517 (59.6)		553 (61.1)	316 (66.5)	237 (55.1)	
1 = moderate-severe	638 (28.2)	267 (21.7)	371 (36.1)		638 (34.8)	288 (29.8)	350 (40.4)		352 (38.9)	159 (33.5)	193 (44.9)	
Difficulty in daily work due to physical health, *n* (%)				<0.001				<0.001				0.012
0 = not at all-somewhat	1937 (85.7)	1091 (88.5)	846 (82.3)		1492 (81.4)	822 (85.1)	670 (77.3)		737 (81.4)	402 (84.6)	335 (77.9)	
1 = quite a lot and above	324 (14.3)	142 (11.5)	182 (17.7)		341 (18.6)	144 (14.9)	197 (22.7)		168 (18.6)	73 (15.4)	95 (22.1)	
Traumatic events, *n* (%)				0.799				0.204				0.370
0 = No	203 (9.4)	109 (9.2)	94 (9.6)		155 (8.9)	74 (8.0)	81 (9.9)		79 (9.2)	46 (10.2)	33 (8.1)	
1 = Yes	1958 (90.6)	1075 (90.8)	883 (90.4)		1587 (91.1)	848 (92.0)	739 (90.1)		779 (90.8)	407 (89.8)	372 (91.9)	
Detention events, *n* (%)				<0.001				<0.001				<0.001
0 = No	1968 (89.0)	991 (82.6)	977 (96.5)		1619 (90.8)	795 (85.1)	824 (97.1)		800 (91.2)	390 (85.7)	410 (97.2)	
1 = Yes	244 (11.0)	209 (17.4)	35 (3.5)		164 (9.2)	139 (14.9)	25 (2.9)		77 (8.8)	65 (14.3)	12 (2.8)	
Refugee camps events, n (%)				<0.001				<0.001				0.029
0 = No	1826 (81.8)	949 (78.1)	877 (86.3)		1472 (81.7)	743 (78.6)	729 (85.2)		708 (79.8)	356 (76.9)	352 (83.0)	
1 = Yes	405 (18.2)	266 (21.9)	139 (13.7)		329 (18.3)	202 (21.4)	127 (14.8)		179 (20.2)	107 (23.1)	72 (17.0)	
Loneliness stressor, *n* (%)				1.000				0.393				0.554
0 = No	1811 (82.1)	995 (82.1)	816 (82.0)		1496 (85.0)	784 (84.2)	712 (85.8)		698 (84.0)	369 (84.8)	329 (83.1)	
1 = Yes	396 (17.9)	217 (17.9)	179 (18.0)		265 (15.0)	147 (15.8)	118 (14.2)		133 (16.0)	66 (15.2)	67 (16.9)	
Number of social integration stressors, *n* (%)				0.004				0.498				0.667
0	837 (37.0)	490 (39.7)	347 (33.8)		1292 (70.5)	688 (71.2)	604 (69.7)		624 (69.0)	331 (69.7)	293 (68.1)	
≥1	1424 (63.0)	743 (60.3)	681 (66.2)		541 (29.5)	278 (28.8)	263 (30.3)		281 (31.0)	144 (30.3)	137 (31.9)	
Family conflicts in Australia, *n* (%)				0.030				1.000				0.013
0	2203 (97.4)	1210 (98.1)	993 (96.6)		1795 (97.9)	946 (97.9)	849 (97.9)		860 (95.0)	460 (96.8)	400 (93.0)	
≥1	58 (2.6)	23 (1.9)	35 (3.4)		38 (2.1)	20 (2.1)	18 (2.1)		45 (5.0)	15 (3.2)	30 (7.0)	
School or study stressor, *n* (%)				0.001				<0.001				0.001
0 = No	1852 (83.9)	1045 (86.2)	807 (81.1)		1569 (89.1)	855 (91.8)	714 (86.0)		786 (94.6)	423 (97.2)	363 (91.7)	
1 = Yes	355 (16.1)	167 (13.8)	188 (18.9)		192 (10.9)	76 (8.2)	116 (14.0)		45 (5.4)	12 (2.8)	33 (8.3)	
Number of family concern stressors, *n* (%)				0.402				0.009				0.252
0	1674 (74.0)	917 (74.4)	757 (73.6)		1240 (67.6)	684 (70.8)	556 (64.1)		607 (67.1)	330 (69.5)	277 (64.4)	
1	397 (17.6)	221 (17.9)	176 (17.1)		448 (24.4)	212 (21.9)	236 (27.2)		237 (26.2)	114 (24.0)	123 (28.6)	
2	190 (8.4)	95 (7.7)	95 (9.2)		145 (7.9)	70 (7.2)	75 (8.7)		61 (6.7)	31 (6.5)	30 (7.0)	
Number of financial hardships, *n* (%)				0.184				0.001				0.054
0	1250 (57.8)	707 (59.8)	543 (55.5)		1196 (66.6)	670 (70.6)	526 (62.1)		544 (62.9)	307 (67.0)	237 (58.2)	
1	395 (18.3)	212 (17.9)	183 (18.7)		270 (15.0)	135 (14.2)	135 (15.9)		111 (12.8)	52 (11.4)	59 (14.5)	
2	267 (12.3)	134 (11.3)	133 (13.6)		155 (8.6)	66 (7.0)	89 (10.5)		88 (10.2)	44 (9.6)	44 (10.8)	
≥3	250 (11.6)	130 (11.0)	120 (12.3)		175 (9.7)	78 (8.2)	97 (11.5)		122 (14.1)	55 (12.0)	67 (16.5)	
COVID-19 stressors, *n* (%)												0.148
0 = No	NA	NA	NA		NA	NA	NA		685 (82.4)	367 (84.4)	318 (80.3)	
1 = Yes	NA	NA	NA		NA	NA	NA		146 (17.6)	68 (15.6)	78 (19.7)	
Unmet support or help during COVID-19, *n* (%)												0.006
1 = never	NA	NA	NA		NA	NA	NA		365 (42.3)	214 (47.9)	151 (36.3)	
2 = rarely	NA	NA	NA		NA	NA	NA		219 (25.4)	101 (22.6)	118 (28.4)	
3 = sometimes	NA	NA	NA		NA	NA	NA		159 (18.4)	72 (16.1)	87 (20.9)	
4 = often-very often	NA	NA	NA		NA	NA	NA		120 (13.9)	60 (13.4)	60 (14.4)	

*Notes:* PTSD: post-traumatic stress disorder; HR-SMI: high risk for severe mental illness; NA: not applicable. *P* values were calculated by chi-square tests to examine subgroup difference; Fisher’s exact test was applied when expected cell counts were <5 (i.e., Region of origin). Numbers might not add up to the column total because of missing data.

**Figure 2.**
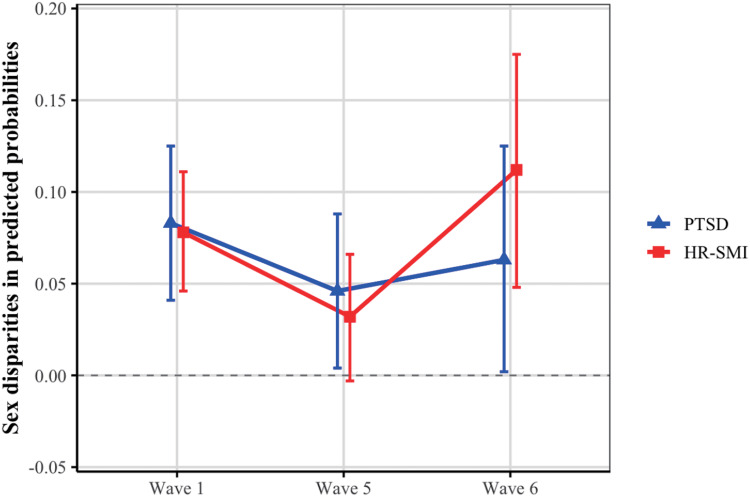
Trajectories of sex disparities in predicted probabilities of PTSD and HR-SMI. PTSD: post-traumatic stress disorder; HR-SMI: high risk for severe mental illness. Sex disparities were calculated as the difference in weighted predicted probabilities between females and males, with male refugees as the reference group.

Taking male refugees as the reference group, the sex disparity in mental health initially narrows and then widens. Figure 2 shows the sex disparities in predicted probabilities of PTSD and HR-SMI. Across all three waves, female refugees consistently exhibited higher predicted probabilities of both outcomes compared to males. For PTSD, the predicted probability difference (female − male) was 8.3% (95% CI: 4.1%, 12.5%) at Wave 1, 4.6% (95% CI: 0.4%, 8.8%) at Wave 5 and 6.3% (95% CI: 0.2%, 12.5%) at Wave 6. For HR-SMI, the difference was 7.8% (95% CI: 4.6%, 11.1%) at Wave 1, 3.2% (95% CI: −0.3%, 6.6%) at Wave 5 and 11.2% (95% CI: 4.8%, 17.5%) at Wave 6. Notably, the magnitude of sex disparities for both PTSD and HR-SMI decreased from Waves 1 to 5 but subsequently increased from Waves 5 to 6.

**Figure 3.**
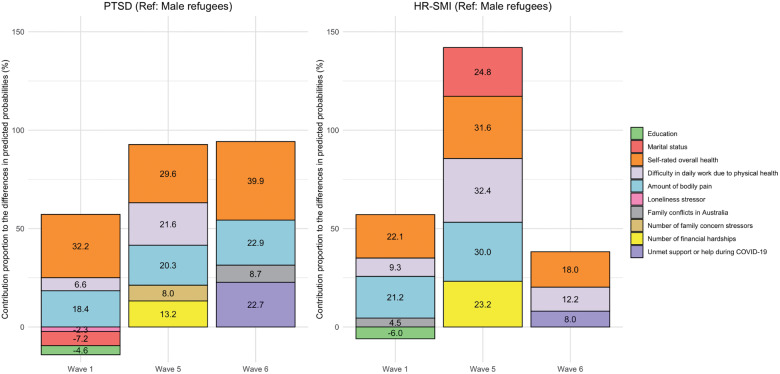
Contribution proportions of significant determinants of the mental health disparity between male and female refugees**.** PTSD: post-traumatic stress disorder; HR-SMI: high risk for severe mental illness; contribution proportion = endowment effect of the variable / total predicted probability difference × 100%. Only determinants that significantly contributed to the disparities are shown in the figure.

At Waves 1, 5 and 6, the endowment effect of all explanatory variables accounted for 44.2%, 103.9% and 125.6% (overall contribution proportion) of sex disparity in PTSD and 55.8%, 160.8% and 67.6% for HR-SMI, respectively. For example, at Wave 5, the sex disparity in PTSD was 4.6%, with an overall contribution proportion exceed 100% (103.9%). This can be explained as if the distribution of all included determinants were equal between females and males, the predicted sex disparity would decrease to −0.2% (contribution proportion × current sex disparity, i.e., −3.9% × 4.6%), implying a 0.2% higher predicted PTSD prevalence among males than females.

Physical health-related factors, including self-rated overall health, amount of bodily pain and difficulty in daily work due to physical health, consistently contributed to the disparities both in PTSD (overall contribution proportion of physical health-related factors was 57.2% at Wave 1; 71.5% at Wave 5; 63.0% at Wave 6) and HR-SMI (52.7% at Wave 1; 93.9% at Wave 5; 41.6% at Wave 6). Additionally, unmet support or help during COVID-19 significantly emerged as a key predictor of sex disparity both in PTSD (22.7%) and HR-SMI (8.0%) at Wave 6.

Some determinants only played important roles at certain stages of resettlement. For PTSD, marital status (contribution proportion, −7.2%), education level (−4.6%) and loneliness stressor (−2.3%) contributed the most to the sex disparity in the initial resettlement stage (Wave 1). For example, if females had the same marital status distribution as males, the sex disparity in PTSD would increase by 0.6% (7.2% × 8.3%). Number of financial hardships (13.2%) and number of family concern stressors (8.0%) significantly contributed to the sex disparity at the middle stage of resettlement (Wave 5). In this case, eliminating the observed sex disparities in financial hardships and family concern stressors would reduce the sex disparity in PTSD to 86.9% and 92.0% of the present disparity, respectively. Family conflicts in Australia accounted for 8.7% of the sex disparity at the long-term stage of resettlement (Wave 6). For HR-SMI, education level and family conflicts explained −6.0% and 4.5% of the sex disparity at the initial stage, respectively. Marital status (24.8%) and number of financial hardships (23.2%) explained the sex disparity at the middle stage of resettlement.

Sensitivity analyses were conducted using complete case dataset and unweighted multiple imputation dataset (Supplementary materials 2 and 3). The complete case results were largely consistent with the main analysis, supporting the robustness of the findings. In contrast, the unweighted multiple imputation analyses showed notable deviations, underscoring the importance of applying sample weights and suggesting that the BNLA weights substantially improve the representativeness of the estimates (Jann, 2023).

## Discussion

Although significant sex disparities in mental health outcomes among resettled refugees have been reported, data examining how such disparities and their determinants evolve over time remains scarce. This study sought to examine determinants and changes in mental health disparities over time by sex among refugees in Australia. The novelty of this study lies in identifying whether, when and to what extent sex disparities emerge and persist over the first 10 years of resettlement and in quantifying key determinants. This resettlement window incidentally included the COVID-19 pandemic, offering an additional universal stressor to contextualize patterns of mental health disparities observed over the decade.

Female refugees generally showed poorer mental health during the resettlement process (for PTSD, the predicted probability difference ranged from 4.6% to 8.3%; for HR-SMI, the difference ranged from 3.2% to 11.2%), with the disparity initially decreasing and then increasing, potentially influenced by the pandemic. Our findings align with other longitudinal studies (Handiso *et al.*, 2024), reporting a higher symptom burden among females, a pattern also observed in the general host population (Olff, 2017). Systematic reviews and meta-analyses have consistently reported that mood disorders (depression and anxiety) are generally more common among female refugees, with the possible exception of PTSD (Lindert *et al.*, 2009; Bogic *et al.*, 2015; Tinghög *et al.*, 2017). The inconsistency in PTSD between this study and previous systematic reviews may be due to various reasons. Firstly, one theoretical interpretation suggests sex differences among refugees are closely related to stigma and report bias. Male refugees tend to under-report PTSD symptoms linked to emotional vulnerability or trauma, such as sexual violence or helplessness compared to females. The under-reporting by males is tied to stigma surrounding mental health, cultural norms and most importantly a desire to maintain the traditional masculine role (Tolin and Foa, 2006; Polusny *et al.*, 2014). Male refugees may also exhibit PTSD via externalizing behaviours – such as emotional dysregulation, anger, interpersonal issues and substance use – that do not meet classic diagnostic criteria, leading to under-recognition of typical PTSD profiles (Rosenfield, 1999; DeSantis *et al.*, 2025). These sex-linked cultural norms and differentials in PTSD symptom expressions cannot be accurately captured by screening tools. Sampling biases further amplify the impact of non-sex-sensitive instruments, as males are less likely to participate in mental health surveys (Smith *et al.*, 2018; Blom *et al.*, 2024; Sheikh *et al.*, 2025). However, in the BNLA survey, visa allocation was linked to the PA, who is usually male, resulting in a higher proportion of male participation than other studies. In the analysis, we also adopted a survey weight to adjust the representativeness of the study sample.

In terms of determinants of the disparities, we found that physical health-related factors made the largest contributions to sex disparities in mental health throughout the first decade of the resettlement process, accounting for more than 50% of the predicted sex disparities in prevalence of PTSD and HR-SMI. Female refugees often carry the long-term consequences of past trauma, chronic conditions and unmet healthcare needs from their countries of origin and during transit (Chalouhi *et al.*, 2025). Conditions such as chronic pain, untreated injuries and poor reproductive health can significantly impair their daily functioning and capacity to work, thereby exacerbating financial instability and compounding psychological distress (Brodda Jansen, 2020; MedCentral, 2022; Nissen *et al.*, 2022). In addition to poor physical health, there are distinct factors that contribute at various stages of resettlement. This pattern aligns with Giddens’ structuration theory, which conceptualizes such disparities as the outcome of the dynamic interplay between sex-related agency and unequal structural conditions (Giddens, 1984). At the initial resettlement stage, demographic characteristics, such as education level and marital status, played more important roles than resettlement-related stressors (e.g., family conflicts in Australia and loneliness). In the BNLA study, the Wave 1 survey was conducted within 1 year after resettlement, indicating resettlement-related stressors, though important (Chen *et al.*, 2017), have similar impacts on mental health of both females and males. In summary, the abovementioned findings suggested, during the initial stage of resettlement, priority services should focus on physical health-related factors through the provision of high-quality, trauma-informed care to reduce sex disparities in mental health. However, it should also be noted that, the variables included in this study explained only a limited proportion of the observed sex disparities at Wave 1, accounting for 44.2% of the disparity in PTSD and 55.8% of the disparity in HR-SMI, suggesting that future research needs to further collect other possible explanatory factors, such as health status and stress factors at the pre-settlement stage.

At the middle stage of resettlement, marital status, financial hardships and family concern stressors were the most important predictors of sex disparities, aside from physical health. In the long-term stage, family conflict again emerged as a key predictor of sex disparities, with its continued influence possibly reflecting broader patterns reported elsewhere of heightened family conflict during the COVID-19 pandemic (Lo *et al.*, 2025). In this study, female refugees experienced more financial hardships, family concern stressors and family conflicts than their male counterparts. There are many factors that predispose this phenomenon, including a combination of factors that encompass the lack of recognition for their skills and qualifications, discrimination and barriers to accessing social support services such as childcare, the burden of parenting and caregiving responsibilities driven by cultural expectations, and limited respite care (Women’s Refugee Commission, 2021; Ziersch *et al.*, 2023; Flavel *et al.*, 2024; Mumtaz *et al.*, 2025). In addition, findings by Handiso and colleagues suggested that females’ mental health is more vulnerable to financial stressors, as they reported greater levels of PTSD and distress compared to males in similar financial hardship conditions (Handiso *et al.*, 2024). This vulnerability may be rooted in social roles and social expectations that systematically constrain females’ access to financial opportunities and resources, thereby amplifying the psychological impact of financial hardship (Hollander *et al.*, 2011; Van Droogenbroeck *et al.*, 2018; Jarallah and Baxter, 2019). These findings demonstrate that the importance of sex-sensitive social services to promote health equity gradually becomes more prominent during the later stages of resettlement. Finally, overall, the COVID-19 pandemic disproportionately affected the mental health among females through increased vulnerability and widening sex inequalities (Arilha *et al.*, 2024). The impact appeared to be greater among refugee females, potentially due to their higher baseline of social adversities, trauma exposures and prevalence of mental disorders (Rees *et al.*, 2023). The pandemic likely amplified the impact of poor physical health, as disruptions to healthcare services may have delayed essential treatments and health support for female refugees, worsening both their physical and mental conditions (Georgetown Institute for Women, Peace and Security, 2021; El Tatary and Gill, 2022).

### Policy implications and limitations

Our findings support that female refugees experience heightened social adversities, cumulative trauma and higher rates of mental disorders, underscoring the need to co-ordinate policy throughout different resettlement stages and at both individual and systemic levels. In the early stage of resettlement, ensuring timely access to culturally appropriate and sex-sensitive physical and mental healthcare is essential. Prioritizing female providers (e.g., clinicians and interpreters), incorporating peer-navigation models of care and culturally competent care models (Alsamman *et al.*, 2025), reducing invisible practical and social barriers such as transport cost, inflexible appointment times and digital exclusion embedding refugee women in service governance can help shift systems towards equitable, preventive and rights-based care. This should continue as resettlement progresses, in addition to strengthening women’s agency through culturally safe, community-led approaches. Long-term economic assistance and supports, such as housing assistance, financial counselling, job training and implementing universal healthcare programs, have been shown to improve access for marginalized populations (Esi Mansa Aidoo, 2023). Advocacy for survivors of intimate partner violence can further promote mental health equity (Rivas *et al.*, 2015; Brooks *et al.*, 2022). Finally, during public health emergencies, such as COVID-19, ensuring continuity of sex-sensitive care, accessible information and enhanced protections for women is vital, as vulnerabilities often intensify during crises.

Despite these findings, there are some limitations worth outlining. Firstly, although the PTSD-8 and Kessler-6 scales are widely used and validated instruments for assessing PTSD symptoms and psychological distress, they are screening tools rather than diagnostic instruments. Therefore, the prevalence of mental health issues among males and females could be overestimated. However, the inflation of prevalence estimates arising from the use of screening tools may be partly attenuated for PTSD among male refugees because of male-specific reporting biases and non-sex-sensitive instruments (Rosenfield, 1999). Consequently, the magnitude of the observed sex disparities in PTSD may have been overestimated and should therefore be interpreted with caution. Secondly, although the twofold Fairlie method allows quantification of group differences and factor contributions, it is limited to measured variables and focuses solely on the endowment effects. Due to limitations in the information available in the BNLA dataset, we were unable to evaluate the contributions of some important unmeasured factors, such as pre-settlement health status and the COVID-19 infection status during the pandemic. Moreover, given the nature of the twofold Fairlie method (Fairlie, 2005), we could not meaningfully interpret the unexplained part, including the coefficient effects. Thirdly, while using decompositions at multiple time points helps us examine the composition of sex disparities in mental health at each stage, this method does not allow for an assessment of how changes in characteristics or their effects over time contribute to the evolution of these disparities. There is a need for the development of a Fairlie method suitable for longitudinal data in the future. Finally, as this study is based exclusively on quantitative analyses, qualitative data could be needed in further studies.

## Conclusion

Our research found that female refugees consistently face greater challenges in mental health than their male counterparts at various stages of resettlement. Poor physical health was a consistently strong determinant to this sex disparity, while family conflict in the host country, financial hardships and family concerns played different roles at the middle and long-term stages of resettlement. In the long-term stage, unmet support or help during the COVID-19 pandemic emerged as a notable factor for the sex disparity. These findings underscore the need for sustained and more targeted policies to reduce mental health inequities among refugee populations. Long-term strategies should include improving access to physical healthcare and financial supports and address family-related stressors. During vulnerable periods such as public health emergencies, more attention should be paid to women’s psychosocial needs to ensure that needs are identified and met.

## Supporting information

10.1017/S2045796026100638.sm001 Bu et al. supplementary material Bu et al. supplementary material

## Data Availability

The BNLA datasets are available for authorized people at the DSS Longitudinal Studies Dataverse (Building a New Life in Australia: The Longitudinal Study of Humanitarian Migrants, Release 6.1 (Waves 1-6) – Building a New Life in Australia Dataverse).

## Supplementary material

The supplementary material for this article can be found at https://doi.org/10.1017/S2045796026100638.
