# Supplementary material for: Mental health disparities by sex: unravelling determinants and changes in the refugee resettlement process over a decade
Source: Epidemiol Psychiatr Sci. 2026 Apr 7;35:e23. doi: 10.1017/S2045796026100638 (PMC13122539; doi:10.1017/S2045796026100638)
Supplement: Bu et al. supplementary material [file S2045796026100638sup001.docx]

Contents

[Supplementary materials 1 1](#_Toc220865736)

[STable 1 Characteristics and prevalence of mental illness of the BNLA participants at three survey waves 1](#_Toc220865737)

[Supplementary materials 2: Missing data analyses and sensitivity analyses 3](#_Toc220865738)

[STable 2 Contributions of determinants of the mental health disparity between male and female refugees based on complete case analysis 5](#_Toc220865739)

[STable 3 Baseline characteristics in sex-based subgroups of participants lost to follow-up at Wave 5 and Wave 6 7](#_Toc220865740)

[Supplementary materials 3: Sensitivity analyses of weighted and unweighted datasets 11](#_Toc220865741)

[STable 4 Contributions of determinants of the mental health disparity between male and female refugees based on unweighted multiple imputed datasets 11](#_Toc220865742)

# Supplementary materials 1

### STable 1 Characteristics and prevalence of mental illness of the BNLA participants at three survey waves

|  | Wave 1  (*N* = 2261) | Wave 5  (*N* = 1833) | Wave 6  (*N* = 905) | *p*15 | *p*56 | *p*16 |
| --- | --- | --- | --- | --- | --- | --- |
| HR-SMI, *n* (%) |  |  |  | 1.000 | 0.002 | 0.001 |
| 0 = No | 1875(82.9) | 1520(82.9) | 705(77.9) |  |  |  |
| 1 = Yes | 386(17.1) | 313(17.1) | 200(22.1) |  |  |  |
| PTSD, *n* (%) |  |  |  | 0.001 | 0.101 | 0.342 |
| 0 = No | 1505(66.6) | 1311(71.5) | 619(68.4) |  |  |  |
| 1 = Yes | 756(33.4) | 522(28.5) | 286(31.6) |  |  |  |
| Sex, *n* (%) |  |  |  | 0.255 | 0.948 | 0.315 |
| 1 = Male | 1233(54.5) | 966(52.7) | 475(52.5) |  |  |  |
| 2 = Female | 1028(45.5) | 867(47.3) | 430(47.5) |  |  |  |
| Age (years), *n* (%) |  |  |  | <0.001 | <0.001 | <0.001 |
| ≤34 | 984(46.3) | 687(37.5) | 212(23.4) |  |  |  |
| 35-49 | 736(34.6) | 657(35.8) | 317(35.0) |  |  |  |
| 50-64 | 315(14.8) | 351(19.1) | 262(29.0) |  |  |  |
| ≥65 | 92(4.3) | 138(7.5) | 114(12.6) |  |  |  |
| Education, *n* (%) |  |  |  | 0.375 | 0.248 | 0.655 |
| 0= below undergraduate or tertiary degree | 1874(83.4) | 1535(84.5) | 739(82.7) |  |  |  |
| 1= undergraduate and tertiary degree | 373(16.6) | 282(15.5) | 155(17.3) |  |  |  |
| Marital status, *n* (%) |  |  |  | <0.001 | 0.004 | <0.001 |
| 0 = single | 956(42.3) | 619(33.8) | 256(28.3) |  |  |  |
| 1 = married or co-habiting | 1305(57.7) | 1214(66.2) | 649(71.7) |  |  |  |
| Region of origin, *n* (%) |  |  |  | 0.309 | 0.170 | 0.416 |
| 1 = North Africa and the Middle East | 1271(56.2) | 1088(59.4) | 501(55.4) |  |  |  |
| 2 = South-East Asia | 124(5.5) | 96(5.2) | 64(7.1) |  |  |  |
| 3 = Southern and Central Asia | 779(34.5) | 591(32.2) | 312(34.5) |  |  |  |
| 4 = Sub-Saharan Africa | 81(3.6) | 55(3.0) | 27(3.0) |  |  |  |
| 5 = Other | 6(0.3) | 3(0.2) | 1(0.1) |  |  |  |
| Self-rated overall health, *n* (%) |  |  |  | 0.185 | 0.069 | 0.003 |
| 1 = very poor-fair | 851(37.6) | 728(39.7) | 393(43.4) |  |  |  |
| 2 = good-excellent | 1410(62.4) | 1105(60.3) | 512(56.6) |  |  |  |
| Amount of bodily pain, *n* (%) |  |  |  | <0.001 | 0.040 | <0.001 |
| 0 = none-mild | 1623(71.8) | 1195(65.2) | 553(61.1) |  |  |  |
| 1 = moderate-severe | 638(28.2) | 638(34.8) | 352(38.9) |  |  |  |
| Difficulty in daily work due to physical health, *n* (%) |  |  |  | <0.001 | 1.000 | 0.004 |
| 0 = not at all-somewhat | 1937(85.7) | 1492(81.4) | 737(81.4) |  |  |  |
| 1 = quite a lot and above | 324(14.3) | 341(18.6) | 168(18.6) |  |  |  |
| Traumatic events, *n* (%) |  |  |  | 0.633 | 0.852 | 0.929 |
| 0 = No | 203(9.4) | 155(8.9) | 79(9.2) |  |  |  |
| 1 = Yes | 1958(90.6) | 1587(91.1) | 779(90.8) |  |  |  |
| Detention events, *n* (%) |  |  |  | 0.064 | 0.779 | 0.075 |
| 0 = No | 1968(89.0) | 1619(90.8) | 800(91.2) |  |  |  |
| 1 = Yes | 244(11.0) | 164(9.2) | 77(8.8) |  |  |  |
| Refugee camps events, *n* (%) |  |  |  | 0.958 | 0.255 | 0.208 |
| 0 = No | 1826(81.8) | 1472(81.7) | 708(79.8) |  |  |  |
| 1 = Yes | 405(18.2) | 329(18.3) | 179(20.2) |  |  |  |
| Loneliness stressor, *n* (%) |  |  |  | 0.017 | 0.567 | 0.229 |
| 0 = No | 1811(82.1) | 1496(85.0) | 698(84.0) |  |  |  |
| 1 = Yes | 396(17.9) | 265(15.0) | 133(16.0) |  |  |  |
| Number of social integration stressors, *n* (%) |  |  |  | <0.001 | 0.435 | <0.001 |
| 0 | 837(37.0) | 1292(70.5) | 624(69.0) |  |  |  |
| ≥1 | 1424(63.0) | 541(29.5) | 281(31.0) |  |  |  |
| Family conflicts in Australia, *n* (%) |  |  |  | 0.352 | <0.001 | 0.001 |
| 0 | 2203(97.4) | 1795(97.9) | 860(95.0) |  |  |  |
| ≥1 | 58(2.6) | 38(2.1) | 45(5.0) |  |  |  |
| School or study stressor, *n* (%) |  |  |  | <0.001 | <0.001 | <0.001 |
| 0 = No | 1852(83.9) | 1569(89.1) | 786(94.6) |  |  |  |
| 1 = Yes | 355(16.1) | 192(10.9) | 45(5.4) |  |  |  |
| Number of family concern stressors, *n* (%) |  |  |  | <0.001 | 0.392 | <0.001 |
| 0 | 1674(74.0) | 1240(67.6) | 607(67.1) |  |  |  |
| 1 | 397(17.6) | 448(24.4) | 237(26.2) |  |  |  |
| 2 | 190(8.4) | 145(7.9) | 61(6.7) |  |  |  |
| Number of financial hardships, *n* (%) |  |  |  | <0.001 | 0.002 | <0.001 |
| 0 | 1250(57.8) | 1196(66.6) | 544(62.9) |  |  |  |
| 1 | 395(18.3) | 270(15.0) | 111(12.8) |  |  |  |
| 2 | 267(12.3) | 155(8.6) | 88(10.2) |  |  |  |
| ≥3 | 250(11.6) | 175(9.7) | 122(14.1) |  |  |  |

Notes: PTSD: post-traumatic stress disorder; HR-SMI: high risk for severe mental illness. *P* values were calculated by chi-square tests; Fisher’s exact test was applied when expected cell counts were < 5 (i.e., Region of origin). Numbers might not add up to the column total because of missing data.

# Supplementary materials 2: Missing data analyses and sensitivity analyses

Two approaches were used to assess the missing data mechanism. First, Little’s MCAR test was applied to evaluate whether the data were missing completely at random (MCAR) (Little & Rubin, 2019). The results indicated that the data were not MCAR at Waves 1 (*p* < 0.05) and 5 (*p* < 0.05), while the MCAR assumption held at Wave 6 (*p* = 0.0688). Second, comparisons between complete case dataset and imputed dataset showed no significant differences in the distributions of variables (all *p* > 0.05). This suggests that missingness was related to observed data, supporting the assumption of missing at random (MAR) (Diggle et al., 2002; Tabachnick & Fidell, 2013). Taken together, these findings suggest a MAR mechanism at Waves 1 and 5, and a MCAR mechanism at Wave 6, which is a special case of MAR, thereby justifying the application of multiple imputation in the present study.

Missing explanatory data were handled using multiple imputation by chained equations. A total of m = 10 imputed datasets were generated. The imputation model included all variables used in the analysis. A random seed of 42 was set to ensure reproducibility. For binary variables, imputation was conducted using logistic regression (“logit” method). For ordered categorical variables, such as number of financial hardships and unmet support or help during the COVID-19 pandemic, ordered logistic regression (“ologit” method) was employed.

To test the robustness of the results, complete case analyses (STable 2) were employed to assess sex disparity in PTSD and HR-SMI across Waves 1, 5, and 6. Qualitatively, the significant determinants were consistent with those in the main analysis, except that amount of bodily pain was non-significant for PTSD at Wave 6. In the quantitative comparison, the magnitude and direction of determinants’ contribution remained largely similar between the complete case and imputed analyses. Overall, these sensitivity analyses indicated that the main findings were robust.

Additionally, we conducted analyses by comparing the baseline (Wave 1) characteristics of participants who were lost to follow-up at Wave 5 and Wave 6, stratifying the analysis by sex (STable 3). First, we found males had higher attrition rates than females (23.9% vs. 18.9% at Wave 5, 51.3% vs. 46.3% at Wave 6). Then, we observed significant differences in a few characteristics between male and female participants who lost to follow-up at Wave 5 and Wave 6 respectively. Specifically, compared to males lost to follow-up, females lost to follow-up were significantly more likely to report poor mental health and self-rated overall health, more severe body pain, more social integration stressors and school or study stressors, and were less likely to experience detention events at both Waves 5 and 6. However, similar sex differences in the distribution of these characteristics were also observed in the analytic sample (Table 1 in the manuscript). For example, in the analytic sample, compared to males, females reported poor mental health (PTSD: 37.7% of females vs. 29.8% of males; HR-SMI: 22.3% vs. 12.7%) and self-rated overall health (54.3% vs. 69.1%), more bodily pain (36.1% vs. 21.7%), more school or study-related stressors (18.9% vs. 13.8%) and social integration stressors (66.2% vs. 60.3%), and a lower proportion of having experienced one or more detention events (15.5% vs. 37.3%).

###

### STable 2 Contributions of determinants of the mental health disparity between male and female refugees based on complete case analysis

|  | **Wave 1** | | | | | **Wave 5** | | | | | **Wave 6** | | | | |
| --- | --- | --- | --- | --- | --- | --- | --- | --- | --- | --- | --- | --- | --- | --- | --- |
|  | **Contribution proportion** | **Coefficient** | ***p*** | **LCI** | **UCI** | **Contribution proportion** | **Coefficient** | ***p*** | **LCI** | **UCI** | **Contribution proportion** | **Coefficient** | ***p*** | **LCI** | **UCI** |
| **PTSD** |  |  |  |  |  |  |  |  |  |  |  |  |  |  |  |
| Age | 0.61% | 0.06% | 0.580 | -0.001 | 0.003 | -2.18% | -0.15% | 0.321 | -0.005 | 0.001 | -3.57% | -0.34% | 0.329 | -0.010 | 0.003 |
| Education | -4.87% | -0.46% | 0.011 | -0.008 | -0.001 | -1.32% | -0.09% | 0.688 | -0.005 | 0.004 | -0.27% | -0.03% | 0.927 | -0.006 | 0.005 |
| Marital status | -5.16% | -0.48% | 0.022 | -0.009 | -0.001 | -3.06% | -0.21% | 0.637 | -0.011 | 0.007 | 1.40% | 0.13% | 0.800 | -0.009 | 0.012 |
| Region of origin | 3.94% | 0.37% | 0.209 | -0.002 | 0.009 | 3.53% | 0.25% | 0.221 | -0.001 | 0.006 | 6.18% | 0.58% | 0.203 | -0.003 | 0.015 |
| Self-rated overall health | 27.79% | 2.60% | 0.000 | 0.018 | 0.034 | 21.43% | 1.51% | 0.000 | 0.008 | 0.022 | 27.42% | 2.58% | 0.001 | 0.011 | 0.041 |
| Difficulty in daily work due to physical health | 6.93% | 0.65% | 0.002 | 0.002 | 0.011 | 18.58% | 1.31% | 0.001 | 0.005 | 0.021 | 5.53% | 0.52% | 0.308 | -0.005 | 0.015 |
| Amount of bodily pain | 17.90% | 1.68% | 0.000 | 0.008 | 0.026 | 12.39% | 0.87% | 0.039 | 0.000 | 0.017 | 12.00% | 1.13% | 0.123 | -0.003 | 0.026 |
| Traumatic events | -0.41% | -0.04% | 0.650 | -0.002 | 0.001 | -2.23% | -0.16% | 0.116 | -0.004 | 0.000 | -0.50% | -0.05% | 0.789 | -0.004 | 0.003 |
| Detention events | -1.21% | -0.11% | 0.784 | -0.009 | 0.007 | -3.74% | -0.26% | 0.644 | -0.014 | 0.009 | -0.91% | -0.09% | 0.938 | -0.022 | 0.021 |
| Refugee camps events | -4.15% | -0.39% | 0.120 | -0.009 | 0.001 | 3.24% | 0.23% | 0.167 | -0.001 | 0.006 | 1.41% | 0.13% | 0.629 | -0.004 | 0.007 |
| Loneliness stressor | -2.96% | -0.28% | 0.004 | -0.005 | -0.001 | -0.36% | -0.03% | 0.764 | -0.002 | 0.001 | 0.46% | 0.04% | 0.823 | -0.003 | 0.004 |
| Number of social integration stressors | 2.90% | 0.27% | 0.073 | 0.000 | 0.006 | -1.06% | -0.07% | 0.676 | -0.004 | 0.003 | -0.90% | -0.08% | 0.729 | -0.006 | 0.004 |
| Family conflicts in Australia | 1.70% | 0.16% | 0.200 | -0.001 | 0.004 | -0.71% | -0.05% | 0.517 | -0.002 | 0.001 | 5.32% | 0.50% | 0.041 | 0.000 | 0.010 |
| School or study stressor | 0.44% | 0.04% | 0.768 | -0.002 | 0.003 | 3.10% | 0.22% | 0.343 | -0.002 | 0.007 | 2.73% | 0.26% | 0.498 | -0.005 | 0.010 |
| Number of family concern stressors | 0.14% | 0.01% | 0.867 | -0.001 | 0.002 | 6.09% | 0.43% | 0.048 | 0.000 | 0.009 | -1.98% | -0.19% | 0.512 | -0.007 | 0.004 |
| Number of financial hardships | 1.47% | 0.14% | 0.259 | -0.001 | 0.004 | 10.02% | 0.70% | 0.016 | 0.001 | 0.013 | 10.01% | 0.94% | 0.050 | 0.000 | 0.019 |
| COVID-19 stressors | NA | NA | NA | NA | NA | NA | NA | NA | NA | NA | 1.56% | 0.15% | 0.507 | -0.003 | 0.006 |
| Unmet support or help during COVID-19 | NA | NA | NA | NA | NA | NA | NA | NA | NA | NA | 16.04% | 1.51% | 0.001 | 0.006 | 0.024 |
| **HR-SMI** |  | | | | |  | | | | |  | | | | |
| Age | -0.40% | -0.03% | 0.805 | -0.003 | 0.002 | -1.46% | -0.06% | 0.702 | -0.004 | 0.003 | -0.60% | -0.07% | 0.831 | -0.007 | 0.005 |
| Education | -7.06% | -0.61% | 0.015 | -0.011 | -0.001 | 0.15% | 0.01% | 0.975 | -0.004 | 0.004 | -0.52% | -0.06% | 0.720 | -0.004 | 0.003 |
| Marital status | 1.10% | 0.10% | 0.562 | -0.002 | 0.004 | 24.17% | 1.00% | 0.013 | 0.002 | 0.018 | 7.83% | 0.87% | 0.060 | 0.000 | 0.018 |
| Region of origin | 4.04% | 0.35% | 0.263 | -0.003 | 0.010 | -2.46% | -0.10% | 0.400 | -0.003 | 0.001 | 1.03% | 0.11% | 0.707 | -0.005 | 0.007 |
| Self-rated overall health | 21.79% | 1.90% | 0.000 | 0.010 | 0.028 | 23.76% | 0.99% | 0.000 | 0.004 | 0.015 | 15.86% | 1.77% | 0.015 | 0.003 | 0.032 |
| Difficulty in daily work due to physical health | 10.79% | 0.94% | 0.000 | 0.005 | 0.014 | 29.93% | 1.24% | 0.001 | 0.005 | 0.020 | 10.73% | 1.20% | 0.014 | 0.002 | 0.021 |
| Amount of bodily pain | 18.13% | 1.58% | 0.001 | 0.006 | 0.025 | 27.22% | 1.13% | 0.001 | 0.005 | 0.018 | 9.46% | 1.05% | 0.147 | -0.004 | 0.025 |
| Traumatic events | -0.60% | -0.05% | 0.590 | -0.002 | 0.001 | 0.07% | 0.00% | 0.966 | -0.001 | 0.001 | 0.29% | 0.03% | 0.826 | -0.003 | 0.003 |
| Detention events | 0.36% | 0.03% | 0.923 | -0.006 | 0.007 | 3.75% | 0.16% | 0.646 | -0.005 | 0.008 | -7.36% | -0.82% | 0.460 | -0.030 | 0.014 |
| Refugee camps events | -5.14% | -0.45% | 0.074 | -0.009 | 0.000 | 2.78% | 0.12% | 0.326 | -0.001 | 0.003 | 0.94% | 0.10% | 0.688 | -0.004 | 0.006 |
| Loneliness stressor | -1.66% | -0.14% | 0.276 | -0.004 | 0.001 | -0.26% | -0.01% | 0.927 | -0.002 | 0.002 | -0.10% | -0.01% | 0.955 | -0.004 | 0.004 |
| Number of social integration stressors | -0.09% | -0.01% | 0.950 | -0.003 | 0.002 | -0.82% | -0.03% | 0.807 | -0.003 | 0.002 | 0.36% | 0.04% | 0.882 | -0.005 | 0.006 |
| Family conflicts in Australia | 5.05% | 0.44% | 0.010 | 0.001 | 0.008 | 2.05% | 0.09% | 0.386 | -0.001 | 0.003 | 2.58% | 0.29% | 0.262 | -0.002 | 0.008 |
| School or study stressor | -0.06% | -0.01% | 0.956 | -0.002 | 0.002 | 2.74% | 0.11% | 0.495 | -0.002 | 0.004 | 2.75% | 0.31% | 0.369 | -0.004 | 0.010 |
| Number of family concern stressors | -0.68% | -0.06% | 0.511 | -0.002 | 0.001 | -0.36% | -0.01% | 0.946 | -0.004 | 0.004 | 0.09% | 0.01% | 0.960 | -0.004 | 0.004 |
| Number of financial hardships | 0.71% | 0.06% | 0.701 | -0.003 | 0.004 | 17.68% | 0.73% | 0.003 | 0.003 | 0.012 | 7.26% | 0.81% | 0.075 | -0.001 | 0.017 |
| COVID-19 stressors | NA | NA | NA | NA | NA | NA | NA | NA | NA | NA | 2.97% | 0.33% | 0.254 | -0.002 | 0.009 |
| Unmet support or help during COVID-19 | NA | NA | NA | NA | NA | NA | NA | NA | NA | NA | 10.26% | 1.14% | 0.009 | 0.003 | 0.020 |

Notes: PTSD: post-traumatic stress disorder; HR-SMI: high risk for severe mental illness; Contribution proportion = endowment effect of the variable / total predicted probability difference × 100%; LCI: Lower limit of the 95% confidence interval; UCI: Upper limit of the 95% confidence interval. Cross-sectional weights were used in the Fairlie method.

### STable 3 Baseline characteristics in sex-based subgroups of participants lost to follow-up at Wave 5 and Wave 6

|  | Wave 5 | | | Wave 6 | | |
| --- | --- | --- | --- | --- | --- | --- |
|  | Male | Female | *p* | Male | Female | *p* |
|  | (*N*=331) | (*N*=228) |  | (*N*=650) | (*N*=503) |  |
| HR-SMI, *n* (%) |  |  | 0.003 |  |  | <0.001 |
| 0 = No | 279.5(89.0) | 166.4(78.0) |  | 555.7(88.7) | 387.1(79.9) |  |
| 1 = Yes | 34.5(11.0) | 47.1(22.0) |  | 71.0(11.3) | 97.1(20.1) |  |
| PTSD, *n* (%) |  |  | <0.001 |  |  | <0.001 |
| 0 = No | 246.5(81.3) | 127.2(61.4) |  | 459.5(75.2) | 291.5(62.8) |  |
| 1 = Yes | 56.5(18.7) | 80.0(38.6) |  | 151.5(24.8) | 172.5(37.2) |  |
| Age (years), *n* (%) |  |  | 0.709 |  |  | 0.746 |
| ≤34 | 214.7(64.9) | 139.4(61.2) |  | 381.7(58.7) | 288.3(57.3) |  |
| 35-49 | 75.2(22.7) | 56.5(24.8) |  | 176.0(27.1) | 145.3(28.9) |  |
| 50-64 | 34.6(10.4) | 28.9(12.7) |  | 66.1(10.2) | 54.2(10.8) |  |
| ≥65 | 6.6(2.0) | 3.0(1.3) |  | 26.5(4.1) | 15.5(3.1) |  |
| Education, *n* (%) |  |  | 0.458 |  |  | 0.194 |
| 0= below undergraduate or tertiary degree | 244.4(75.2) | 177.5(78.6) |  | 520.7(81.0) | 419.5(84.5) |  |
| 1= undergraduate and tertiary degree | 80.7(24.8) | 48.4(21.4) |  | 122.1(19.0) | 77.1(15.5) |  |
| Marital status, *n* (%) |  |  | 0.209 |  |  | 0.273 |
| 0 = single | 150.0(45.3) | 117.5(51.6) |  | 281.0(43.2) | 235.0(46.7) |  |
| 1 = married or co-habiting | 181.0(54.7) | 110.3(48.4) |  | 369.4(56.8) | 268.2(53.3) |  |
| Region of origin, *n* (%) |  |  | 0.001 |  |  | 0.003 |
| 1 = North Africa and the Middle East | 120.6(36.5) | 109.0(48.7) |  | 324.3(49.9) | 271.2(54.5) |  |
| 2 = South-East Asia | 37.5(11.4) | 28.2(12.6) |  | 44.7(6.9) | 43.9(8.8) |  |
| 3 = Southern and Central Asia | 147.9(44.8) | 55.9(25.0) |  | 251.3(38.7) | 139.3(28.0) |  |
| 4 = Sub-Saharan Africa | 24.0(7.3) | 30.5(13.6) |  | 29.1(4.5) | 42.9(8.6) |  |
| Self-rated overall health, *n* (%) |  |  | <0.001 |  |  | <0.001 |
| 1 = very poor-fair | 58.0(17.5) | 76.2(33.4) |  | 152.0(23.4) | 195.1(38.8) |  |
| 2 = good-excellent | 272.9(82.5) | 151.7(66.6) |  | 498.5(76.6) | 308.1(61.2) |  |
| Amount of bodily pain, *n* (%) |  |  | <0.001 |  |  | <0.001 |
| 0 = none-mild | 282.0(85.2) | 158.8(69.7) |  | 538.9(82.9) | 340.5(67.7) |  |
| 1 = moderate-severe | 49.0(14.8) | 69.1(30.3) |  | 111.5(17.1) | 162.8(32.3) |  |
| Difficulty in daily work due to physical health, *n* (%) |  |  | 0.132 |  |  | 0.003 |
| 0 = not at all-somewhat | 298.4(90.2) | 194.5(85.4) |  | 589.5(90.6) | 425.9(84.6) |  |
| 1 = quite a lot and above | 32.6(9.8) | 33.4(14.6) |  | 60.9(9.4) | 77.3(15.4) |  |
| Traumatic events, *n* (%) |  |  | 0.604 |  |  | 0.395 |
| 0 = No | 41.7(13.4) | 24.3(11.6) |  | 67.2(11.1) | 60.0(13.0) |  |
| 1 = Yes | 269.3(86.6) | 185.8(88.4) |  | 540.2(88.9) | 401.4(87.0) |  |
| Detention events, *n* (%) |  |  | <0.001 |  |  | <0.001 |
| 0 = No | 252.4(79.0) | 209.9(94.8) |  | 511.5(81.9) | 470.4(96.1) |  |
| 1 = Yes | 67.1(21.0) | 11.5(5.2) |  | 113.1(18.1) | 18.9(3.9) |  |
| Refugee camps events, *n* (%) |  |  | 0.132 |  |  | 0.008 |
| 0 = No | 250.7(77.1) | 183.3(83.5) |  | 485.7(76.6) | 411.4(83.9) |  |
| 1 = Yes | 74.3(22.9) | 36.2(16.5) |  | 148.8(23.4) | 78.8(16.1) |  |
| Loneliness stressor, *n* (%) |  |  | 0.886 |  |  | 0.882 |
| 0 = No | 258.8(83.4) | 174.8(83.9) |  | 513.4(82.5) | 399.8(82.8) |  |
| 1 = Yes | 51.5(16.6) | 33.5(16.1) |  | 109.1(17.5) | 82.9(17.2) |  |
| Number of social integration stressors, *n* (%) |  |  | 0.034 |  |  | 0.006 |
| 0 | 195.0(58.9) | 110.4(48.4) |  | 315.4(48.5) | 199.1(39.6) |  |
| ≥1 | 135.9(41.1) | 117.5(51.6) |  | 335.0(51.5) | 304.2(60.4) |  |
| Family conflicts in Australia, *n* (%) |  |  | 0.057 |  |  | 0.251 |
| 0 | 324.8(98.1) | 216.0(94.8) |  | 635.7(97.7) | 485.5(96.5) |  |
| ≥1 | 6.2(1.9) | 11.8(5.2) |  | 14.8(2.3) | 17.8(3.5) |  |
| School or study stressor, *n* (%) |  |  | 0.008 |  |  | 0.017 |
| 0 = No | 263.2(84.8) | 153.5(73.7) |  | 542.5(87.1) | 393.0(81.4) |  |
| 1 = Yes | 47.1(15.2) | 54.8(26.3) |  | 80.0(12.9) | 89.7(18.6) |  |
| Number of family concern stressors, *n* (%) |  |  | 0.948 |  |  | 0.907 |
| 0 | 250.0(75.5) | 169.0(74.2) |  | 484.3(74.5) | 375.8(74.7) |  |
| 1 | 57.3(17.3) | 41.7(18.3) |  | 118.5(18.2) | 94.0(18.7) |  |
| 2 | 23.6(7.1) | 17.1(7.5) |  | 47.6(7.3) | 33.5(6.7) |  |
| Number of financial hardships, *n* (%) |  |  | 0.641 |  |  | 0.494 |
| 0 | 165.2(53.0) | 116.5(53.5) |  | 363.1(58.8) | 255.5(53.8) |  |
| 1 | 52.7(16.9) | 42.7(19.6) |  | 103.6(16.8) | 88.0(18.5) |  |
| 2 | 38.6(12.4) | 18.9(8.7) |  | 72.6(11.7) | 61.5(12.9) |  |
| ≥3 | 55.0(17.7) | 39.9(18.3) |  | 78.5(12.7) | 70.2(14.8) |  |

Notes: PTSD: post-traumatic stress disorder; HR-SMI: high risk for severe mental illness; *P* values were calculated by chi-square tests to examine subgroup difference; Fisher’s exact test was applied when expected cell counts were < 5 (i.e., Region of origin). In the attrition sample, categories of region of origin with zero or one observation were collapsed for analysis. Numbers might not add up to the column total because of missing data. Cross-sectional weights were used to adjust the representativeness.

# Supplementary materials 3: Sensitivity analyses of weighted and unweighted datasets

Analysis with unweighted imputed datasets was conducted to compare with results in Figure 3 using weighted imputed datasets. Results were shown in STable 4, which yielded notably different results for region of origin (Wave 1), refugee camps events (Wave 5), marital status (Wave 6), and number of financial hardships (Waves 1, 5 and 6), highlighting discrepancies from the main analysis. This underscored the importance of weighting, as the BNLA weights appear to be informative rather than ignorable.

### STable 4 Contributions of determinants of the mental health disparity between male and female refugees based on unweighted multiple imputed datasets

|  | **Wave 1** | | | | | **Wave 5** | | | | | **Wave 6** | | | | |
| --- | --- | --- | --- | --- | --- | --- | --- | --- | --- | --- | --- | --- | --- | --- | --- |
|  | **Contribution proportion** | **Coefficient** | ***p*** | **LCI** | **UCI** | **Contribution proportion** | **Coefficient** | ***p*** | **LCI** | **UCI** | **Contribution proportion** | **Coefficient** | ***p*** | **LCI** | **UCI** |
| **PTSD** | | | | | | | | | | | | | | | |
| Age | 0.07% | 0.01% | 0.905 | -0.001 | 0.001 | 0.05% | 0.00% | 0.978 | -0.002 | 0.002 | 0.09% | 0.01% | 0.975 | -0.004 | 0.004 |
| Education | -3.95% | -0.33% | 0.003 | -0.005 | -0.001 | 1.19% | 0.05% | 0.750 | -0.003 | 0.004 | -1.48% | -0.09% | 0.592 | -0.004 | 0.002 |
| Marital status | -10.19% | -0.85% | 0.001 | -0.014 | -0.003 | -1.18% | -0.05% | 0.869 | -0.007 | 0.006 | 4.26% | 0.27% | 0.599 | -0.007 | 0.013 |
| Region of origin | 7.56% | 0.63% | 0.002 | 0.002 | 0.010 | 0.35% | 0.02% | 0.875 | -0.002 | 0.002 | 4.37% | 0.28% | 0.137 | -0.001 | 0.006 |
| Self-rated overall health | 32.24% | 2.68% | 0.000 | 0.019 | 0.034 | 38.53% | 1.78% | 0.000 | 0.011 | 0.025 | 36.51% | 2.30% | 0.000 | 0.013 | 0.033 |
| Difficulty in daily work due to physical health | 10.65% | 0.89% | 0.000 | 0.005 | 0.013 | 21.46% | 0.99% | 0.001 | 0.004 | 0.016 | 0.83% | 0.05% | 0.861 | -0.005 | 0.006 |
| Amount of bodily pain | 21.44% | 1.78% | 0.000 | 0.009 | 0.027 | 23.36% | 1.08% | 0.002 | 0.004 | 0.018 | 18.45% | 1.16% | 0.022 | 0.002 | 0.022 |
| Traumatic events | -0.47% | -0.04% | 0.578 | -0.002 | 0.001 | -0.86% | -0.04% | 0.405 | -0.001 | 0.001 | 0.07% | 0.00% | 0.940 | -0.001 | 0.001 |
| Detention events | -5.24% | -0.44% | 0.340 | -0.013 | 0.005 | 10.13% | 0.47% | 0.244 | -0.003 | 0.013 | 3.14% | 0.20% | 0.714 | -0.009 | 0.013 |
| Refugee camps events | -2.57% | -0.21% | 0.330 | -0.006 | 0.002 | 8.60% | 0.40% | 0.011 | 0.001 | 0.007 | 7.51% | 0.47% | 0.054 | 0.000 | 0.010 |
| Loneliness stressor | -0.36% | -0.03% | 0.490 | -0.001 | 0.001 | -0.36% | -0.02% | 0.575 | -0.001 | 0.000 | 0.65% | 0.04% | 0.627 | -0.001 | 0.002 |
| Number of social integration stressors | 1.91% | 0.16% | 0.147 | -0.001 | 0.004 | -1.52% | -0.07% | 0.226 | -0.002 | 0.000 | -1.24% | -0.08% | 0.475 | -0.003 | 0.001 |
| Family conflicts in Australia | 0.52% | 0.04% | 0.623 | -0.001 | 0.002 | -0.06% | 0.00% | 0.883 | 0.000 | 0.000 | 10.48% | 0.66% | 0.008 | 0.002 | 0.012 |
| School or study stressor | -1.40% | -0.12% | 0.256 | -0.003 | 0.001 | 6.66% | 0.31% | 0.123 | -0.001 | 0.007 | -4.54% | -0.29% | 0.351 | -0.009 | 0.003 |
| Number of family concern stressors | 1.16% | 0.10% | 0.098 | 0.000 | 0.002 | 9.07% | 0.42% | 0.021 | 0.001 | 0.008 | 0.99% | 0.06% | 0.750 | -0.003 | 0.004 |
| Number of financial hardships | 2.57% | 0.21% | 0.041 | 0.000 | 0.004 | 8.30% | 0.38% | 0.079 | 0.000 | 0.008 | 15.62% | 0.99% | 0.005 | 0.003 | 0.017 |
| COVID-19 stressors | NA | NA | NA | NA | NA | NA | NA | NA | NA | NA | 2.37% | 0.15% | 0.249 | -0.001 | 0.004 |
| Unmet support or help during COVID-19 | NA | NA | NA | NA | NA | NA | NA | NA | NA | NA | 27.79% | 1.75% | 0.000 | 0.010 | 0.025 |
| **HR-SMI** | | | | | | | | | | | | | | | |
| Age | -0.35% | -0.03% | 0.789 | -0.002 | 0.002 | 3.15% | 0.10% | 0.324 | -0.001 | 0.003 | 0.51% | 0.05% | 0.772 | -0.003 | 0.004 |
| Education | -5.19% | -0.41% | 0.011 | -0.007 | -0.001 | 1.42% | 0.05% | 0.799 | -0.003 | 0.004 | -1.45% | -0.15% | 0.390 | -0.005 | 0.002 |
| Marital status | 1.07% | 0.08% | 0.697 | -0.003 | 0.005 | 30.11% | 0.96% | 0.006 | 0.003 | 0.016 | 10.65% | 1.11% | 0.030 | 0.001 | 0.021 |
| Region of origin | 2.93% | 0.23% | 0.133 | -0.001 | 0.005 | -4.79% | -0.15% | 0.153 | -0.004 | 0.001 | 0.80% | 0.08% | 0.534 | -0.002 | 0.003 |
| Self-rated overall health | 20.41% | 1.60% | 0.000 | 0.009 | 0.023 | 42.52% | 1.35% | 0.000 | 0.008 | 0.019 | 16.82% | 1.75% | 0.001 | 0.007 | 0.028 |
| Difficulty in daily work due to physical health | 13.01% | 1.02% | 0.000 | 0.006 | 0.015 | 33.65% | 1.07% | 0.000 | 0.006 | 0.016 | 10.39% | 1.08% | 0.002 | 0.004 | 0.018 |
| Amount of bodily pain | 26.75% | 2.10% | 0.000 | 0.013 | 0.029 | 35.68% | 1.13% | 0.000 | 0.006 | 0.017 | 9.87% | 1.03% | 0.050 | 0.000 | 0.021 |
| Traumatic events | -0.16% | -0.01% | 0.856 | -0.001 | 0.001 | 0.16% | 0.01% | 0.875 | -0.001 | 0.001 | 0.00% | 0.00% | 0.995 | -0.001 | 0.001 |
| Detention events | 5.19% | 0.41% | 0.134 | -0.001 | 0.009 | 6.18% | 0.20% | 0.496 | -0.004 | 0.008 | -2.29% | -0.24% | 0.653 | -0.013 | 0.008 |
| Refugee camps events | -4.09% | -0.32% | 0.164 | -0.008 | 0.001 | 5.20% | 0.17% | 0.086 | 0.000 | 0.004 | 2.22% | 0.23% | 0.332 | -0.002 | 0.007 |
| Loneliness stressor | 2.15% | 0.17% | 0.066 | 0.000 | 0.003 | -0.09% | 0.00% | 0.960 | -0.001 | 0.001 | 1.51% | 0.16% | 0.200 | -0.001 | 0.004 |
| Number of social integration stressors | 0.04% | 0.00% | 0.973 | -0.002 | 0.002 | -0.43% | -0.01% | 0.764 | -0.001 | 0.001 | -0.12% | -0.01% | 0.922 | -0.003 | 0.002 |
| Family conflicts in Australia | 5.19% | 0.41% | 0.000 | 0.002 | 0.006 | 0.03% | 0.00% | 0.973 | -0.001 | 0.001 | 5.17% | 0.54% | 0.057 | 0.000 | 0.011 |
| School or study stressor | 0.40% | 0.03% | 0.653 | -0.001 | 0.002 | 6.68% | 0.21% | 0.146 | -0.001 | 0.005 | 0.99% | 0.10% | 0.755 | -0.005 | 0.008 |
| Number of family concern stressors | -1.30% | -0.10% | 0.218 | -0.003 | 0.001 | 1.38% | 0.04% | 0.793 | -0.003 | 0.004 | 1.64% | 0.17% | 0.432 | -0.003 | 0.006 |
| Number of financial hardships | 3.18% | 0.25% | 0.027 | 0.000 | 0.005 | 18.93% | 0.60% | 0.002 | 0.002 | 0.010 | 10.00% | 1.04% | 0.004 | 0.003 | 0.017 |
| COVID-19 stressors | NA | NA | NA | NA | NA | NA | NA | NA | NA | NA | 0.07% | 0.01% | 0.958 | -0.003 | 0.003 |
| Unmet support or help during COVID-19 | NA | NA | NA | NA | NA | NA | NA | NA | NA | NA | 9.47% | 0.99% | 0.005 | 0.003 | 0.017 |

Notes: PTSD: post-traumatic stress disorder; HR-SMI: high risk for severe mental illness; Contribution proportion = endowment effect of the variable / total predicted probability difference × 100%; LCI: Lower limit of the 95% confidence interval; UCI: Upper limit of the 95% confidence interval. Fairlie method was employed.

# References

**Diggle PJ, Heagerty PJ, Liang K and Zeger SL** (2002) Analysis of Longitudinal Data. *Oxford University Press*

**Little R and Rubin D** (2019) Statistical Analysis with Missing Data, Third Edition. *Wiley*

**Tabachnick B and Fidell L** (2013) Using Multivariate Statistics. *Pearson International*
